# Targeting alternative splicing in cancer immunotherapy

**DOI:** 10.3389/fcell.2023.1232146

**Published:** 2023-08-10

**Authors:** Nan Han, Zhaoqi Liu

**Affiliations:** ^1^ Chinese Academy of Sciences Key Laboratory of Genomic and Precision Medicine, Beijing Institute of Genomics, Chinese Academy of Sciences and China National Center for Bioinformation, Beijing, China; ^2^ University of Chinese Academy of Sciences, Beijing, China

**Keywords:** alternative splicing, immunotherapy, cancer, organoid, precision medicine

## Abstract

Tumor immunotherapy has made great progress in cancer treatment but still faces several challenges, such as a limited number of targetable antigens and varying responses among patients. Alternative splicing (AS) is an essential process for the maturation of nearly all mammalian mRNAs. Recent studies show that AS contributes to expanding cancer-specific antigens and modulating immunogenicity, making it a promising solution to the above challenges. The organoid technology preserves the individual immune microenvironment and reduces the time/economic costs of the experiment model, facilitating the development of splicing-based immunotherapy. Here, we summarize three critical roles of AS in immunotherapy: resources for generating neoantigens, targets for immune-therapeutic modulation, and biomarkers to guide immunotherapy options. Subsequently, we highlight the benefits of adopting organoids to develop AS-based immunotherapies. Finally, we discuss the current challenges in studying AS-based immunotherapy in terms of existing bioinformatics algorithms and biological technologies.

## 1 Introduction

Immunotherapy harnesses the immune system to eliminate tumor cells through various methods, including immune checkpoint blockade (ICB), monoclonal antibody (mAb), adoptive cell therapy (ACT), and cancer vaccines (Jain, 2021; Zhu et al., 2021). ICB eliminates cancer cells by binding endogenous immunosuppressive molecules via the antibodies, reactivating exhausted CD8^+^ cytotoxic T lymphocytes (Topalian et al., 2015). Monoclonal antibodies, ACT, and cancer vaccines are primarily designed based on cancer-specific antigens (Scott et al., 2012; Weiner, 2015). The mAb is derived from a single B-cell clone, engineered to recognize tumor cells through antigen-antibody interactions. When utilizing mAbs in oncology, several mechanisms of action exist to destroy the cancer cells (Bayer, 2019), such as blocking cellular processes (Keam, 2020), flagging cells for an immune attack (Czuczman and Gregory, 2010; Goldsmith et al., 2021), or inducing cell death when mAb is paired with a cytotoxic agent (Thomas et al., 2016). ACT, specifically chimeric antigen receptor (CAR) T-cell immunotherapy, is a highly personalized immunotherapy. Patient-derived T cells are modified to carry a CAR that targets a specific tumor antigen. Once modified, these CAR T-cells are reinfused into the patient to target the tumor cells via CAR and eradicate them through a cytotoxic reaction (Zhang et al., 2017). Cancer vaccines contain external immunoreactive components like neoantigen peptides, nucleic acids, or immunomodulatory agents. Instead of targeting and killing tumors, these substances activate anti-tumor immunity through indirect ways, including increasing the production of tumor-specific antigens and introducing cytokines (Morse et al., 2021; Saxena et al., 2021). These immunotherapies have proven to be a success in treating some types of hematological and solid cancers, especially melanoma (Okazaki et al., 2013; Weiss et al., 2019). A recent study showed that immunotherapy improved the median survival of a subset of patients with advanced melanoma from approximately 6 months to nearly 6 years (Knight et al., 2023). However, they still face difficulties, such as a limited number of immunogenic antigens for selection (Kirkwood et al., 2009; Lawson et al., 2015) and a high rate of treatment resistance (Bagchi et al., 2021).

Most current studies focus on somatic mutation-derived neoantigens or treatment resistance, while the potential role of splicing in this process is often overlooked. Alternative splicing is required for the maturation of mammalian mRNAs in that pre-mRNA introns are removed and various exons are selected and joined, generating diverse transcripts and proteins from the same DNA template (Wang et al., 2008; Nilsen and Graveley, 2010). As reported, more than 95% of human genes undergo pre-mRNA splicing (Pan et al., 2008; Ajith et al., 2016; Baralle and Giudice, 2017; Zhang et al., 2020) and splicing dysregulation has been detected in many cancers (Climente-González et al., 2017; Kahles et al., 2018). Trans-regulator disruptions, such as splicing factors mutations (Harbour et al., 2013; Dvinge et al., 2016) and cis-element changes, are frequently occurred in tumors, which contribute to a wide range of splicing dysregulation and influence tumor formation, tumor metastasis and treatment response (Wu et al., 2023).

First, mis-splicing has been shown to play a role in tumor initiation (Gaur et al., 2008; Stavik et al., 2013; Koh et al., 2015; Shirai et al., 2015; Pellagatti et al., 2018; Huan et al., 2020). For instance, c-Myc increases the transcription of splicing regulators PTB, hnRNPA1, and hnRNPA2, leading to an alternative isoform switch from *PKM1* to *PKM2* that affect the cancer initiation via cell metabolism (David et al., 2010; Yang et al., 2012; Ma et al., 2022). In addition, mis-splicing of tumor suppressor genes, such as *BRCA1, PTEN* in breast cancer (Okumura et al., 2011), and *KRAS* in lung cancer (Pio and Montuenga, 2009; Nussinov et al., 2016; Aran et al., 2018), are also reported to promote tumor initiation (Jung et al., 2015; Venkataramany et al., 2022). Second, oncogenes have also been shown to undergo isoforms switching as a mechanism for cancer cells to metastasize (Choi et al., 2019; Inoue et al., 2019; Fish et al., 2021). *CD44* produces variant (CD44v) isoforms by alternative splicing of variant exons. These CD44v isoforms are highly expressed in metastatic tumors, and promote epithelial-to-mesenchymal transition and cell invasion (Brown et al., 2011; Chen et al., 2018). Moreover, there are other isoforms have been reported to promote tumor metastasis, including KLF6-SV1 (DiFeo et al., 2009; Hatami et al., 2013), and BRCA1-IRIS (Bogan et al., 2017) in breast cancer. Third, mis-splicing can also contribute to cancer treatment resistance (Mitra et al., 2009; Palladini et al., 2017; Hsu et al., 2020). For example, the HER2D16 splicing variant is highly expressed in a subset of HER2+ breast cancer patients with resistance to trastuzumab, a HER2-targeted therapy (Palladini et al., 2017; Hsu et al., 2020). Similarly, one BRAF(V600E) splicing variant lacks the binding domain of the RAF inhibitor vemurafenib, leading to acquired drug resistance in melanoma patients with *BRAF* mutations (Poulikakos et al., 2011). Besides the above splicing variants, other cancer-associated splicing events have been summarized in Table 1.

**TABLE 1:** Summary of cancer-associated splicing isoforms.

Cancer	Gene	Type	Biological function
LC	*KRAS*	ES	K-RAS4A mediates the oncogenic activity of K-Ras in carcinogenesis. Pio and Montuenga (2009); Nussinov et al. (2016); Aran et al. (2018)
LC	*BCL2L1*	ES	Bcl-xL promotes cell survival, tumorigenesis. Bcl-xS promotes apoptosis. Boise et al. (1993)
LC	*ERBB2*	ES	HER2D16 mediates Osimertinib resistance in lung cancer. Hsu et al. (2020)
LC	*CEACAM1*	ES	CEACAM-1L and CEACAM-1S mediate the apoptosis pathway. Nittka et al. (2008); Gonzalez-Exposito et al. (2019)
BC	*PLEC*	ES	SNRPA1 regulates the splicing of *PLEC and* enhances tumor invasion. Fish et al. (2021)
BC	*HER2*	ES	HER2D16 mediates tumorigenesis and HER2-targeted therapy resistance. Hsu et al. (2020)
BC	*KLF6*	ES	KLF6-SV1 is related to breast cancer metastasis and poor survival. DiFeo et al. (2009); Hatami et al. (2013)
BC	*CEACAM1*	ES	The ratio of S:L isoforms of *CEACAM1* may mediate tumorigenesis. Gaur et al. (2008)
LK	*TMPO*	ES	The MYC protein helps with RNA splicing in lymphomagenesis by producing specific anti-proliferative and apoptotic isoforms through PRMT5 in acute lymphoblastic leukemia. Koh et al. (2015)
LK	*LEF1*	ES	
LK	*HDAC7*	ES	
LK	*NTAN1*	ES	
LK	*POMT1*	ES	
LK	*BCL2*	ES	Bcl-2α is an anti-apoptotic protein contributing to tumorigenesis. Boise et al. (1993)
LK	*WT1*	A5SS	Increased expression of the WT1-KTS is associated with poor prognosis. (Ullmark et al. (2017)
LK	*INTS3*	IR	Mis-splicing of *INTS3*, contributing to leukemogenesis. Yoshimi et al. (2019)
LK	*CD33*	ES	SNP in the splicing enhancer region regulates the expression of D2-CD33, which leads to resistance to gemtuzumab ozogamicin. Lamba et al. (2017)
LK	*IRAK4*	ES	U2AF1 mutations induced IRAK4-L to promote tumorigenesis. Smith et al. (2019)
CC	*CEACAM1*	ES	CEACAM1 isoforms are required to inhibit colonic tumor cell growth. Gaur et al. (2008)
GC	*PICALM*	ES	SRSF6 promotes autophagy activity by regulating the *PICALM* exon 14 skipping and triggers a S-to-L isoform switching. Zhang et al. (2021b)
HCC	*EXOC7*	ES	PTBP1 regulates *EXOC7* splicing to control the inflammatory secretome and pro-tumorigenic effects of senescent cells. Georgilis et al. (2018)
HCC	*PXN*	ES	The MBNL3 promotes HCC by increasing *PXN* expression through the alternative splicing of *lncRNA-PXN-AS1.* Yuan et al. (2017)
ML	*PAK1*	ES	JMJD6 promotes melanoma carcinogenesis through the regulation of the AS of *PAK1*, a key MAPK signaling component. Liu et al. (2017b)
ML	*BRAF*	ES	The BRAF(V600 E) splicing variant lacks the RAF inhibitor binding domain, leading to drug resistance in melanoma patients. Poulikakos et al. (2011)
ML	*BRD9*	ES	Mutant SF3B1 induces a poison exon that causes the degradation of *BRD9*, which promotes melanomagenesis. Inoue et al. (2019)
MDS	*EZH2*	ES	SRSF2 mutant cells induce a poison exon resulting in NMD of *EZH2* and impaired hematopoietic differentiation. Kim et al. (2015)
MDS	*CASP8*	ES	SRSF2 Mutations upregulate the CASP8^TR^ isoform, which hyperactivates NF-κB signaling and promotes cell death. Lee et al. (2018)
MDS	*GNAS*	ES	Both mutant U2AF1 and SRSF2 can promote a long *GNAS* isoform, which encodes a more active Gαs protein to activate ERK/MAPK signaling. Wheeler et al. (2022)
MDS	*IRAK4*	ES	U2AF1 mutations induce IRAK4-L*,* activating innate immunity in MDS. Smith et al. (2019)
MDS	*AKAP8*	ES	SRSF2 mutation induces mis-splicing of *AKAP8* to regulate cell growth. Pellagatti et al. (2018)
PDAC	*ARHGAP17*	ES	Alternative splicing of the tumor suppressor *ARHGAP17* increases the GTP hydrolysis of RAS and promotes metastasis. Escobar-Hoyos et al. (2020)
PDAC	*HMMR*	ES	RHAMMB, but not RHAMMA isoform, promotes tumor metastasis. Choi et al. (2019)

Abbreviations: LC, lung cancer; BC, breast cancer; LK, leukemia; CC, colon cancer; CRC, colorectal cancer; GC, gastric cancer; HCC, hepatocellular carcinoma; ML, melanoma; MDS, myelodysplastic syndrome; PDAC, pancreatic ductal adenocarcinoma; UM, uveal melanoma.

Interestingly, recent studies have highlighted a new function of AS in tumors as an important source to expand the pool of neoantigens (Frankiw et al., 2019; Wang et al., 2021) as well as adjusting tumor immune microenvironments (Li et al., 2019; Zhong et al., 2022), which suggests that AS is also implicated in tumor immunotherapy. Currently, the studies of AS-associated immunotherapy rely on time/cost-consuming animal experiments (Ott et al., 2017; Chen et al., 2019; Arnaud et al., 2020; Cheng et al., 2022). Particularly, immunotherapies targeting AS also need suitable models for assessment before clinical. Thus, organoids, a 3D *in vitro* culture system derived from autologous tissue stem cells, may facilitate the development of splicing-based immunotherapies (Xu et al., 2018; Yuki et al., 2020). Numerous studies have demonstrated that organoid technology can provide a high-throughput screening and validation platform to reduce experimental costs and improve validation efficiency (Liu L. et al., 2021; Guillen et al., 2022). Moreover, it can also mimic the *in vivo* microenvironment of the original patient tissue, offering better personalized and rapid models for pre-clinical evaluation (Xu et al., 2018).

In this review, we summarize three critical roles of AS in immunotherapy: neoantigen resources for antigen-based immunotherapy, modulatory targets for adjuvant immunotherapy, and therapeutic biomarkers to guide immunotherapy options. We also highlight the potential advantages of adopting organoids to study splicing-based immunotherapy. Finally, we discuss current challenges in identifying immunotherapy-related AS events and targeting AS in immunotherapy from the perspectives of bioinformatics algorithms and biological technology.

## 2 Section

### 2.1 AS serve as neoantigen resources for antigen-based immunotherapy

The effect of immunotherapy varies across tumor types and patient populations (Okazaki et al., 2013; Weiss et al., 2019; Chamoto et al., 2020), highlighting the necessity of developing personalized immunotherapies based on tumor-specific antigens, which are absent in normal tissues (Jhunjhunwala et al., 2021). Studies have shown that patients with more tumor-specific antigens receive increased sensitivity to neoantigen-based immunotherapies, as well as activate more potent anti-tumor immune responses under ICB therapy. Therefore, individuals with a high mutation burden tend to produce more neoantigens, which makes them benefit more from immunotherapy (Ott et al., 2017; Yarchoan et al., 2019; Blass and Ott, 2021).

In addition to the somatic mutation, aberrant splicing is also an essential origination of neoantigens. Mis-splicing in various tumor types leads to the production of tumor-specific peptides (Frankiw et al., 2019). Previous studies have shown that peptides derived from mis-splicing can bind to major histocompatibility complex class I (MHC I) for T-cell recognition (Jayasinghe et al., 2018; Kahles et al., 2018; Smart et al., 2018; Frankiw et al., 2019). Although most aberrant splicing may introduce early stop codons leading to nonsense-mediated mRNA decay (NMD), some of these RNAs can still undergo a pioneer round of translation to produce some peptides to activate the immune system (Apcher et al., 2011). Compared to mutations that typically affect a single amino acid, mis-splicing, especially intron retention, often inserts a non-coding sequence into the transcript to generate more neoepitopes (Smart et al., 2018). Furthermore, many tumors often accompany the dysfunction of splicing factors (SFs), which exhibit widespread mis-splicing across the whole transcriptome (Wang and Aifantis, 2020). This global splicing change can produce more neoantigens than somatic SNV in many tumor types. For instance, in breast and ovarian cancers, mis-splicing produces at least twice as many neoantigens as those generated by nonsynonymous mutations (Jayasinghe et al., 2018; Kahles et al., 2018).

The splicing dysregulation in tumors can accelerate the development of neoantigen-based immunotherapy, as it provides an expanded candidate pool of antigens for positive selection. For example, fibronectin (FN) encoded by *FN1* is a valuable AS-derived antigen resource (Villa et al., 2008; Huijbers et al., 2010; Jailkhani et al., 2019; Xie et al., 2019; Wagner et al., 2021). Through alternative splicing, *FN1* can generate three distinct adhesive extracellular matrix isoforms, each with unique structural regions: V (IIICS), EIIIA (EDA), and EIIIB (EDB) (Dubin et al., 1995). Studies show that fibronectin containing EDA and EDB segments were significantly upregulated during tumor angiogenesis; while displaying low expression levels in normal adult tissues (Khan et al., 2005; Su et al., 2020). Based on this unique AS pattern, the CAR T-cell, mAbs, and cancer vaccine against EDA or EDB have been developed and shown to reduce tumor growth in several solid tumors, including melanoma and lung adenocarcinoma (Villa et al., 2008; Huijbers et al., 2010; Xie et al., 2019; Wagner et al., 2021). It is worth noting that these two splicing derivatives are accumulated in neovasculature, which is present in most solid tumors. Thus, immunotherapies based on EDA and EDB hold promise for extensive applications across tumors.

*CD44* is another important gene whose AS-derived antigens have been targeted by many immunotherapies. For example, the mAb RG7356 targets the CD44s isoform and has shown efficacy in clinical trials for acute myeloid leukemia (Vey et al., 2016; D'Arena et al., 2014). Another mAb selectively targeting CD44v6 has also shown success in treating many cancers, including squamous cell carcinomas and a subset of adenocarcinomas (Heider et al., 2004). In ACT therapies, CD44v6-targeted CAR-T cells coexpressing a suicide gene eradicate autologous leukemia *in vivo* (Casucci et al., 2013). Besides targeting the FN and CD44 isoform, there also exist many successful immunotherapy designs targeting AS-derived other neoantigens (Table 2), such as a mAb against the splicing structural domain D of tenascin C (Nadal et al., 2020), mAb designed to bind to CLDN18.2 peptide (Sahin et al., 2008), and a cancer vaccination based on D393-CD20 peptide (Vauchy et al., 2015). Although additional function and safety tests are required, these designs hold an excellent prospect for clinical usage.

**TABLE 2:** Summary of recent AS-based studies associated with immunotherapy.

Three roles of AS in immunotherapy	Publication	Experimental model	Main conclusion
Neoantigen resources for antigen-based immunotherapy	Villa et al. (2008)	Mouse model, cell line	Anti-EDA mAb efficiently targets tumor neovasculature *in vivo*
	Xie et al. (2019)	Mouse model, cell line	Anti-EIIIB fibronectin-targeted CAR T-cells slow B16 melanoma growth *in vivo*
	Wagner et al. (2021)	Mouse model, cell line	EDB-CAR T-cells had potent antitumor activity in systemic tumor xenograft models
	Huijbers et al. (2010)	Mouse model, cell line	The vaccination against the EDB domain of FN reduces tumor size in a mouse model
	Nadal et al. (2020)	Mouse model, cell line	A fusion protein against the alternative domain D of Tenascin C exhibited potent antitumor activity in a mouse model
	Sahin et al. (2008)	Mouse model, cell line	A mAb that binds exclusively to the CLDN18.2 isoform is raised and successfully recognizes the antigen on the surface of cancer cells
	Vauchy et al. (2015)	Mouse model, cell line	D393-CD20 peptide-based vaccination can induce specific CD8 and CD4 T cell responses in HLA-humanized transgenic mice
	Vey et al. (2016)	Patient	The mAb RG7356 targets the CD44s isoform and shows efficacy in clinical trials for acute myeloid leukemia
	Heider et al. (2004)	Mouse model	The mAb targeting CD44v6 shows success in treating many cancers
	Casucci et al. (2013)	Mouse model	CD44v6-targeted CAR-T cells mediate potent antitumor effects in myelomas and leukemia
Modulatory targets for adjuvant immunotherapy	Lu et al. (2021)	Mouse model, cell line	Pharmacologic perturbation of SF RBM39 suppresses tumor growth in a manner dependent on host T cells
	Matsushima et al. (2022)	Mouse model, cell line	Regulation of the splicing factor SRSF family boosts immunogenicity and suppresses tumor growth
	Bowling et al. (2021)	Mouse model, cell line	RNA splicing inhibition induces antiviral and adaptive immune signaling in immune-competent models
Therapeutic biomarkers to guide immunotherapy options	Jailkhani et al. (2019)	Mouse model, cell line	The nanobody NJB2 against EDB of the FN domain can detect tumor progression, metastasis, and fibrosis in several solid tumor mouse models
	Fischer et al. (2017)	Cell line	CD19 isoforms resistant to CART-19 immunotherapy are expressed in B-ALL patients at initial diagnosis
	Gong et al. (2019)	Mouse model, cell line	Secreted PD-L1 variants mediate resistance to PD-L1 blockade therapy in non-small cell lung cancer
	Qu et al. (2021)	Mouse model, cell line	PD-L1-lnc increases proliferation and decreases apoptosis of lung adenocarcinoma cells
	Sotillo et al. (2015)	Organoid, mouse model, cell line	A truncated protein of CD19 isoforms provides a proliferative advantage in B-lymphoid cell lines and Xenograft models
	Yang et al. (2022)	Organoid, mouse model, cell line	Tumors with MARCO-TST isoform expression conferred greater sensitivity to treating bromodomain and extraterminal protein inhibitors
	Chandrakesan et al. (2020)	Organoid, mouse model, cell line	DCLK1-isoform2 inhibits CD8^+^ T-cell proliferation and promotes immunosuppressive M2-macrophage polarization in pancreatic tumor
	Zhao et al. (2021)	Cell line	The CD19 ex2part splicing variant represents a new biomarker predictive of blinatumomab therapy failure
	Troiani et al. (2020)	Organoids	Tumor T-cell interaction can induce FKBP51 splicing isoform, which may guide the resistance to ICB therapy
	Weng et al. (2022)	Organoids, cell line	The skipping of exon 17 of TMC7 inhibited the proliferation, invasion, and migration of pancreatic cancer cells
	Chan et al. (2021)	Organoids, cell line	TSLP isoform sfTSLP promoted tumor growth of ovarian and endometrial cancers

**FIGURE 1:**
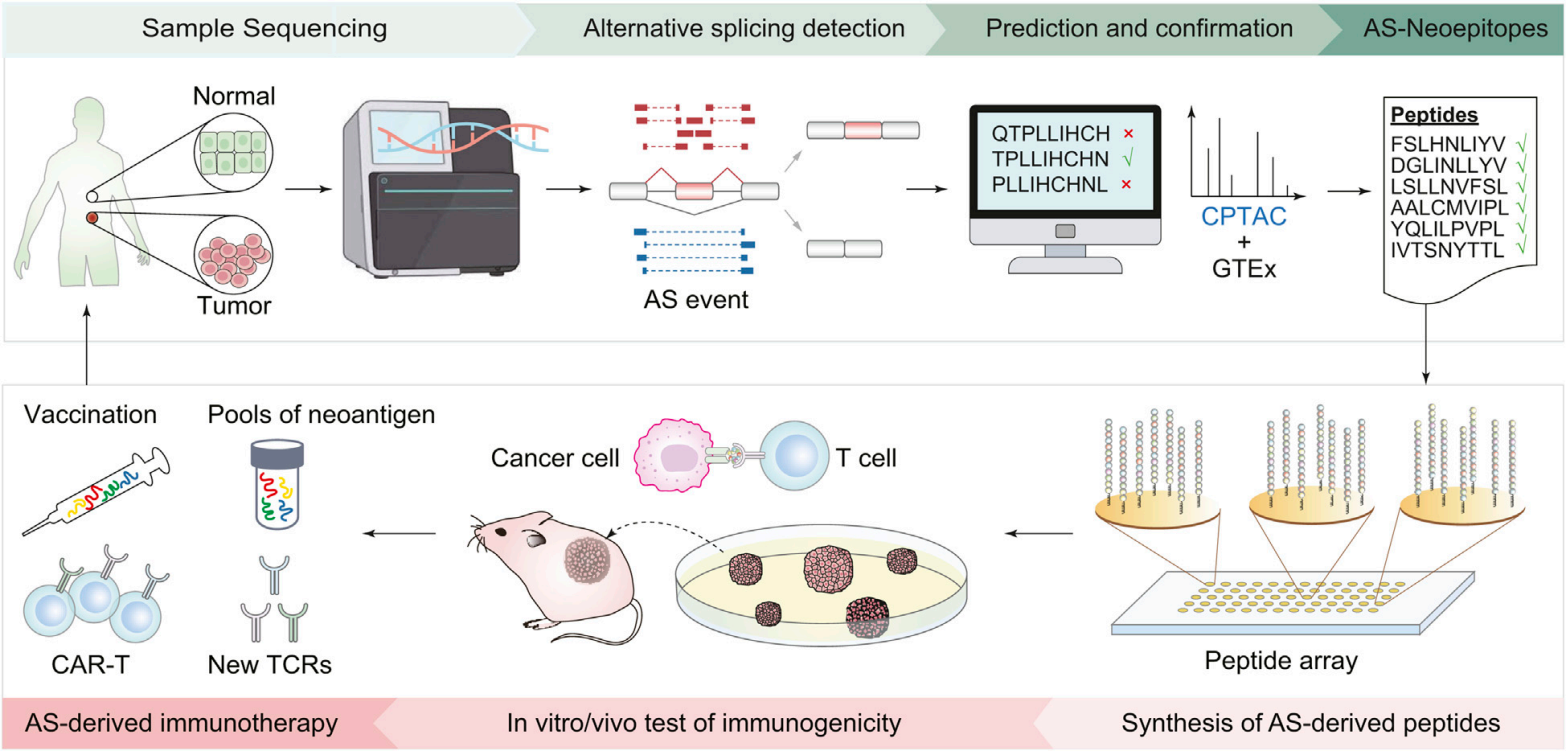
Workflow of immunotherapy design based on AS-derived cancer neoantigens. First, collect tumor and normal tissues from cancer patients and perform transcriptomic sequencing. Bioinformatics tools are then employed to identify AS events, screen peptides derived from AS, and predict their immunogenicity. Based on the list of predicted peptide candidates, peptides are synthesized accordingly. The next steps involve validation experiments both in the cells and living organisms, including using cell lines to assess the stability of peptide-MHC, T-cell function to evaluate the immune response of peptide candidates, and mouse models to check the effect of the AS-derived immunotherapies. Finally, new cancer vaccines and CAR-T cells are designed based on immunogenic AS derivates.

Typically, developing an immunotherapy strategy targeting splicing-derived neoantigen involves the following steps (Figure 1). First, bioinformatics approaches are used to detect cancer-specific AS events, screen AS-derived peptides, and predict the immunogenicity of peptide candidates. Then, based on the prioritized list of peptide candidates, peptides are synthesized to mimic potential immunogenic epitopes. Next, *in vitro* validations, such as peptide-MHC stability assay and T-cell function assay, are conducted to evaluate the immunogenicity of candidate peptides. Finally, *in vivo* validations with animal models are carried out to examine the effect of neoantigen-based immunotherapies. The efficacy of the designed therapy can be evaluated by phenotypes like changes in tumor size and immune cell infiltration (Ott et al., 2017; Chen et al., 2019; Arnaud et al., 2020; Cheng et al., 2022).

Tools have been developed for neoantigen screening by evaluating the binding affinity between splicing-derived peptides and various MHC-I (MHC-II) allotypes (Bayer, 2019). These Peptide-MHC immunogenicity prediction tools generally fall into two categories: scoring-based, for instance, PSSMHCpan (Liu G. et al., 2017) and MixMHCpred (Bassani-Sternberg et al., 2017), and machine learning-based, such as POLYSOLVER (Shukla et al., 2015). Upon extraction of AS-derived antigens that bind strongly to MHC molecules, it is also necessary to use computer tools to assess the immunological activity against the identified antigens. Agent-Based Models (ABMs) are the main computational approaches for such analyses (Butner et al., 2022). In ABM, each cell is represented as a discrete object (agent). These agents interact with their environment following predefined biological rules over discrete time steps (Bonabeau, 2002). One of the most successful ABM methods is IMMSIM (Puzone et al., 2002), which simulates T-cell responses, including T-cell activation, proliferation, differentiation, and antigen recognition. Following this approach (Celada and Seiden, 1992), to date, many ABMs are designed to predict a more comprehensive immune system response, not just the T-cell responses (Celada and Seiden, 1992; Chavali et al., 2008; Pennisi et al., 2013; Madonia et al., 2017; Shou et al., 2022). For example, One ABM model SimB16 was utilized to predict the immune responses of immunotherapy in B16 melanoma (Pappalardo et al., 2011). Another ABM model NetLogo has been successfully adapted to describe the interactions between the immune system and tumor cells (Chiacchio et al., 2014). In addition to ABMs, there are also several alternative approaches, such as Virtual Cell (Resasco et al., 2012) and PySB (Lopez et al., 2013), which use differential equation models to simulate changes in immune cells and cytokines to test the immunological activity. Collectively, these models will greatly speed up the design of immunotherapy targeting splicing-derived neoantigen.

However, the time-consuming development of animal models may not keep pace with the computational identification of antigens for a large-scale candidate screening analysis (Cheng et al., 2022). In future studies, tumor organoids are potential alternative models for the optimization of antigen-based immunotherapy, which will be discussed in subsequent sections.

### 2.2 AS serve as modulatory targets for adjuvant immunotherapy

**FIGURE 2:**
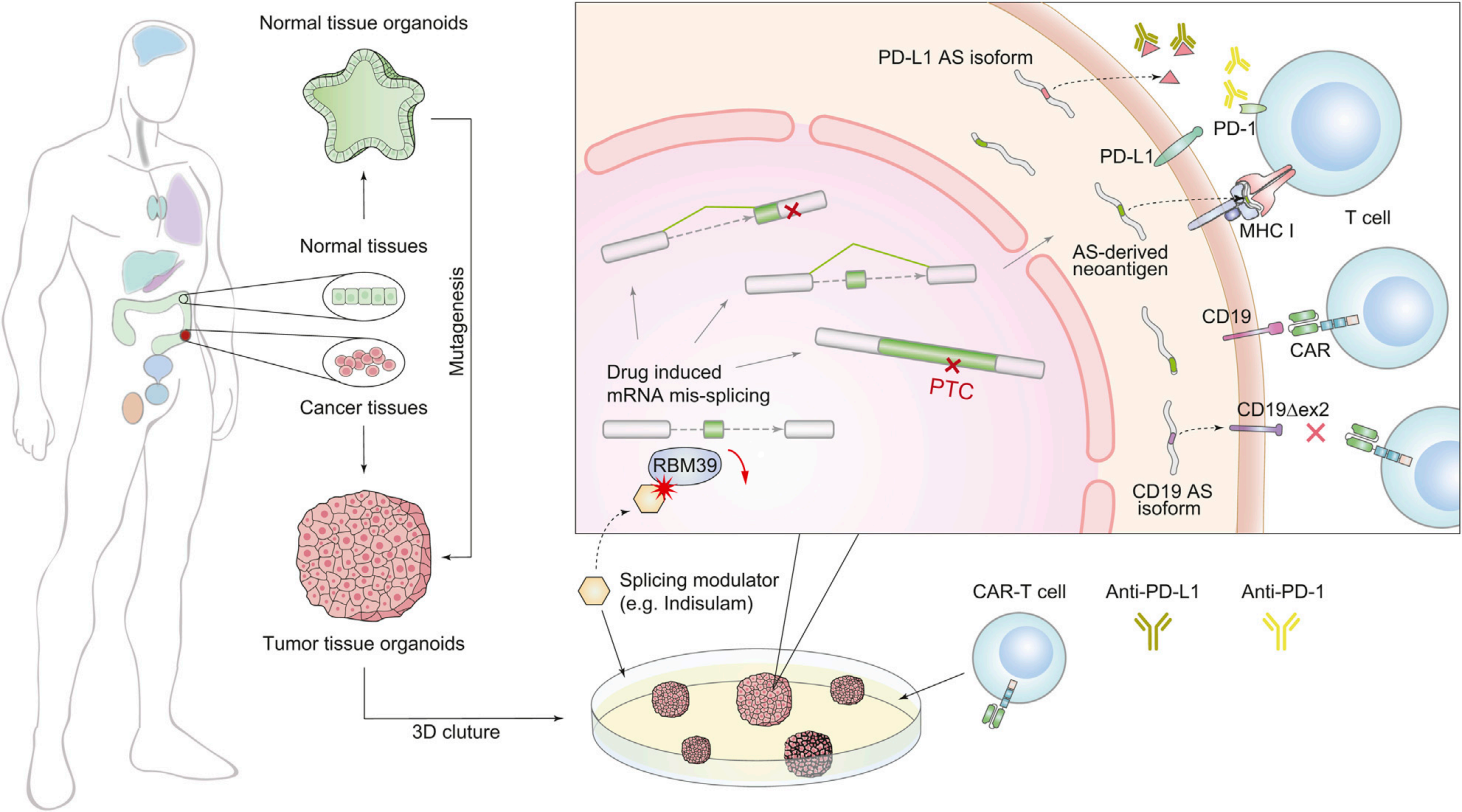
Schematic diagram of combined treatment of immunotherapy and splicing modulations using the organoid system. The organoid culture technology (left) accelerates the combination therapy (right). We generated two forms of splicing modulation as a complementary strategy for immunotherapy. The first one is targeting splicing factors to induce global splicing errors for expanding peptide diversity, e.g., RBM39. The second one is targeting AS derivatives that induce treatment resistance, e.g., PD-L1 and CD19 isoforms.

As discussed above, the dysregulation of SFs may enhance immunogenicity by inducing widespread splicing defects, suggesting that combining immunotherapy with modulations of splicing factors can improve the therapeutic effect of immunotherapy (Figure 2). In mouse models of several solid tumors, degradation of the splicing factor RBM39 generates numerous AS-derived neoantigens, subsequently stimulating anti-tumor immunity and augmenting the effect of ICB therapy (Lu et al., 2021). In another study of colorectal cancer, the chemical activation of the splicing factor SRSF family achieves a similar effect (Matsushima et al., 2022). Furthermore, splicing manipulation can also boost immunogenicity by generating double-stranded RNA (dsRNAs) (Bowling et al., 2021; Ishak et al., 2021). Recent studies have revealed that spliceosome-targeted therapies can cause widespread cytoplasmic accumulation of mis-spliced mRNAs, a significant portion of which can form dsRNAs. These dsRNAs can be recognized by intracellular immune sensors and induce apoptosis in breast cancer cells (Bowling et al., 2021). Although only confirmed in mouse models, these examples provide compelling evidence that splicing modulation can activate immunogenicity and enhance the response to immunotherapy by potentially turning “cold” tumors into “hot” tumors (Galon and Bruni, 2019).

Besides the above contributions of AS-induced immunogenicity, on the other side, many mis-splicing events have also been found to associate with immunotherapy resistance (Gamonet et al., 2015a; Gamonet et al., 2015b; Sotillo et al., 2015; Fischer et al., 2017; Wang and Lee, 2018; Gong et al., 2019; Sciarrillo et al., 2020; Troiani et al., 2020; Deng et al., 2021; Qu et al., 2021; Bourcier and Abdel-Wahab, 2022; Zheng et al., 2022). For example, (Figure 2), in non-small cell lung cancer, secreted PD-L1 splicing isoforms can compete for binding PD-L1 antibodies, resulting in resistance to PD-L1 blockade therapy (Gong et al., 2019). In lung adenocarcinoma, aberrant splicing of the PD-L1 gene can produce a long non-coding RNA (lncRNA), which promotes resistance by enhancing c-Myc activity (Qu et al., 2021). Although CART-19 treatment targeting CD19 achieves a 70% response rate in patients with B cell acute lymphoblastic leukemia, tumors can also evade treatment via generating *CD19* isoforms lacking exon 2 (Sotillo et al., 2015). These observations suggest that targeting harmful AS derivates is a complementary strategy for immunotherapy.

It should be noted that AS-driven resistance to immunotherapy works by different mechanisms. Therefore, the approaches to overcome these resistances vary accordingly (Figure 2). The secreted PD-L1 isoforms lead to drug resistance through competition for PD-L1 antibodies. Thus, using PD-1 antibodies or depleting the AS derivatives by antisense oligonucleotides (ASOs) are practical options (Gong et al., 2019). However, CD19 variants alter the antigen structure and escape from recognition by conventional CART-19. To overcome this resistance, depletion of the CD19 splicing variants cannot rescue the lack of CD19 antigen, and a newly engineered CAR T-cell targeting the CD19 AS-derived antigen is a better choice (Sotillo et al., 2015).

### 2.3 AS serve as therapeutic biomarkers to guide immunotherapy options

Inter-tumor heterogeneity presents a major reason for variable responses to immunotherapy (Saito et al., 2019; Chen et al., 2021). Distinct immune subtypes based on transcriptome successfully predict prognosis and immunotherapy responses in some cancer types (Chen et al., 2021). Alternative splicing is an essential source of transcriptomic and proteomic heterogeneity, which can further help to improve patient stratification. Mis-splicing can remodel the immune microenvironment in tumors. Studies have shown that AS-derived neoantigens can participate in immune reprogramming and directly influence the formation of the tumor microenvironment (TME) (Zhang Y. et al., 2021), suggesting that patients who harbor different splicing substrates may respond differently to the same immunotherapy (Zhang Y. et al., 2021; Su et al., 2022). Another study has validated this hypothesis in lung adenocarcinoma. This study showed that a specific subset of patients with a particular splicing pattern had been found to have higher immunogenicity, leading to better response rates to ICB therapy than other patients (Wu et al., 2022).

Furthermore, splicing alterations typically occur in a cancer-specific or stage-specific manner (Bonnal et al., 2020), indicating that the AS can act as biomarkers to optimize the therapeutic strategy (Le et al., 2015; Chandrakesan et al., 2020; Yang et al., 2022). Mis-splicing can serve as biomarkers for immunotherapy response. Similar to the correlation observed between ICB response and tumor mutational burden, patients with a higher mis-splicing disorder in the tumor tissues may present a higher response rate of ICB therapy (Frankiw et al., 2019; Lu et al., 2021; Matsushima et al., 2022). Besides, another study found that specific AS signatures can indicate immune activity and can be used to predict the response to immunotherapy (Chen et al., 2022). Moreover, some splicing variants can lead to resistance to particular immunotherapy. Besides mentioned AS derivates related to resistance, there are also many other AS events related to resistance, highlighting their critical role as therapeutic biomarkers. For example, skipping exons 5 and 6 of *CD22* leads to resistance to CD22 CAR T-cells (Zheng et al., 2022), and *D393-CD20* can lead to resistance to CD20 mAb therapy (Gamonet et al., 2015b). These observations demonstrate that splicing biomarkers can potentially assist patients in determining immunotherapy choices (Troiani et al., 2020; Qu et al., 2021; Zhao et al., 2021; Weng et al., 2022).

### 2.4 The benefits of adopting organoids in AS-based immunotherapies

In the above studies, the experimental models play a crucial role in screening AS-derived antigens, evaluating AS-related immunotherapy, and exploring the biological functions of AS events. But the commonly used experimental models in current studies are mainly cancer cell lines and mouse models (Table 2), which have some limitations. First, *in vitro* culture of cells cannot accurately replicate the interactions with other cell types or the extracellular matrix. Second, differences in the genome and microenvironment between species make it challenging to translate findings from mice to humans. Especially, the nature of poor conservation of intronic sequences and minimal overlap of mis-spliced transcripts between mice and humans (Lieu et al., 2022) suggest that mice may be less useful in modeling patients with AS dysregulation. Last, it is complex and time-consuming to manipulate genes in mice by multigenerational hybridization, which may result in patients missing optimal treatment periods (Shang et al., 2022; Stribbling and Ryan, 2022). Recent studies have shown that organoids are highly effective in disease modeling and are widely utilized for basic research, drug selection and personalized medicine, which may also benefit the splicing-targeted immunotherapies (Drost and Clevers, 2018).

In cancer modeling, tumor organoids can recapitulate the (epi)genetic and phenotypic diversity of distinct tumor cell subclones, as well as their morphological features (LeSavage et al., 2022). Furthermore, tumor organoids also enable the modeling of TME, including the functions of non-neoplastic cells, the signaling of niche-specific soluble factors, and the altered extracellular matrix (Neal et al., 2018; Yuki et al., 2020). To date, many tumor organoids have been developed and reproduced pathological features (Yeung et al., 2010; Boj et al., 2015; Drost et al., 2016; Hubert et al., 2016). For example, tumor organoids with 3D microfluidic devices maintain immune cell composition of the donor tumors and are used to evaluate the response of ICB treatment (Jenkins et al., 2018). Another approach to culture organoids with an air-liquid interface system achieves similar success (Homicsko, 2020). Additionally, tumor organoids can be grown for an extended period, modified to investigate specific genetic alterations and maintain their features across multiple passages, making them wildly used for basic research.

Tumor organoids are a reliable model for the functional study of alternative splicing events (Rossi et al., 2018; Artegiani et al., 2020; Dekkers et al., 2021). For instance, a study uses cortical organoids to demonstrate that the reintroduction of the archaic splicing variant of NOVA1 alters neurodevelopment (Trujillo et al., 2021). Another example of co-culturing organoids and autologous lymphocytes proved that interactions between melanoma tumor cells and T-cells can induce splicing isoform *FKBP51s*, which is related to the resistance of anti-PD1 blockade therapy (Troiani et al., 2020).

Organoids also provide an ideal model for large-scale drug screening. And specially, drug screening using patient-derived organoids can further guide personalized treatment options. One successful example is ‘eribulin’, selected by cancer xenografts and organoid platforms. Individuals with this treatment achieved complete remission for nearly 5 months (Guillen et al., 2022). More importantly, organoids are valuable for exploring treatment combinations. A previous study has shown that the combination of KRAS inhibitor AMG501 and EGFR inhibitor cetuximab achieves a synergistic effect for treating colorectal cancer organoids with *KRAS* ^G12C^ mutation (Amodio et al., 2020). Another tumor organoid from circulating tumor cells of patients proved that GKB202 is a promising adjuvant for 5-FU-based treatment (Li et al., 2021). Furthermore, the organoid is particularly effective in immuno-related drug selection, as the response to immunotherapy is shaped by both cancer cells and the TME (Xu et al., 2018). To date, many organoid platforms have been developed to evaluate the effects of CAR T-cell therapy (Dijkstra et al., 2018; Schnalzger et al., 2019), ICB therapy (Jenkins et al., 2018), or other neoantigen-based immunotherapies (Courau et al., 2019; Gonzalez-Exposito et al., 2019).

Currently, though, there are no organoid platforms for investigating the functional role of alternative splicing, screening AS-derived neoantigens, or directly evaluating splicing-based immunotherapy. The establishment of these platforms in the future will definitely accelerate the application of alternative splicing in immunotherapy (Raue et al., 2023).

## 3 Discussion

There are questions remain to be addressed in the development of AS-based immunotherapies. Next, we will discuss these challenges in two parts.

### 3.1 Challenges in the identification of AS events associated with cancer immunotherapy

The priority in identifying immunotherapy-related AS derivates is the comprehensive detection of AS events. Numerous computational tools have been developed to detect AS events, yet selecting the optimal algorithm remains challenging due to the large inconsistencies between the outputs of different software (Wang et al., 2015; Mehmood et al., 2020). Given the different designs and sensitivity of algorithms in detecting different types of AS events, one naive way is to integrate the power of multiple algorithms by manually selecting the results, which will induce artificial bias (Mehmood et al., 2020). There is an urgent need for the customized design of more reliable algorithms, considering the specific characteristics of AS events and research requirements. For example, SF3B1 mutation leads to the cryptical 3`splcing sites (3′ss), many of which are not reported in the latest annotation reference. As there is no method specific to detect these 3′ss events, an effective method is needed to expand the reference by creating a dataset-specific annotation file (DeBoever et al., 2015; Liu and Rabadan, 2021).

In addition to algorithm limitations, sequencing technology also encounters obstacles in identifying AS events. Currently, most studies identify and quantify splicing isoforms starting from bulk short-read RNA-seq data (Cieślik and Chinnaiyan, 2018; Ferragut Cardoso et al., 2022; Toffali et al., 2023). These studies typically map the short-read RNA sequences to a reference genome using software such as MISO (Katz et al., 2010) or rMATS (Shen et al., 2014), or assemble *de novo* using tools including StringTie (Pertea et al., 2015) or Trinity (Grabherr et al., 2011). These methods enable the identification of splicing junctions and estimation of isoform abundance based on read counts, providing a global picture of alternative splicing events over the bulk tissue level. However, the short sequencing reads are limited to detect complex and full-length novel isoforms (De Paoli-Iseppi et al., 2021). Additionally, Bulk RNA-seq data is unable to depict intratumor heterogeneity or identify AS events specifically and commonly expressed in all cancer clones, which are the important feature of neoantigen. Single-cell long-read RNA-seq is a powerful tool for studying splicing heterogeneity, while it still has limitations such as low throughput and technical noise. Future efforts should focus on developing more efficient single-cell long-read RNA-seq technology (Singh et al., 2019) and customized algorithms. Finally, the best practices may involve coupled analysis using both long reads and short reads sequencing techniques (Au et al., 2013).

After identifying and quantifying AS events, researchers need to pinpoint AS event candidates contributing to cancer immunotherapy. Many *in silico* tools have been employed to predict immunological activity against new antigens (Rammensee et al., 1993; Lin et al., 2008; Andreatta and Nielsen, 2016; Jurtz et al., 2017) and analyze the function of cancer-related AS events (Kahles et al., 2018; Liu B. et al., 2021; Liu and Rabadan, 2021; Qi et al., 2022). However, experimental validation of the immunogenicity of such computationally predicted neoantigens will need to be seriously assessed. Understanding the function of AS events is often challenging because it is difficult to introduce abnormal AS isoforms into the experimental model. Therefore, many experimental studies are only limited to AS events that lead to NMD, because the loss-of-function consequences are easier to manipulate and interpret (Lareau et al., 2007; Thomas et al., 2020; Bradley and Anczuków, 2023). Excitingly, there are new approaches of DNA/RNA-targeted CAS with CRISPR-based screening to carry out more unbiased analysis of splicing events and their impacts on tumor (Mou et al., 2017). It is worth noting that manipulating DNA sequence may induce off-target effects, such as creating unexpected splice sites and disrupting the chromatin structure.

Moreover, current experiment models in this field are based on cell lines or mouse models, but they harbor limitations as discussed above. Organoids offer an alternative model for investigating AS derivatives and neoantigen screening; however, their application in AS-based immunotherapy is still in its infancy. There is an urgent need to build one-stop organoid platforms for studying splicing-based immunotherapy (Chandrakesan et al., 2020; Yang et al., 2022). It should be noted that all organoids, cell lines, and mouse models possess distinct strengths and limitations. The selection of an appropriate model should be guided by the specific research need.

### 3.2 Challenges in targeting AS in immunotherapy

AS-derived neoantigen may also display a high tumor heterogeneity with a varying distribution in different tumor clones. Immunotherapies based on one single target could only eliminate a part of tumor cells, which may accelerate tumor evolution and disease relapse. Thus, a better option is using multiple-target immunotherapies (such as polyclonal antibodies) or combining multiple therapy strategies, which may cover all cancer clones. Furthermore, some AS products show dramatically higher expression in tumors, which does not mean these AS events are totally absent in normal cells. For example, the reported AS-derived antigens CD44v isoform, CD20 isoform D393-CD20 (Vauchy et al., 2015) and CLDN18 isoform CLDN18.2 (Sahin et al., 2008), are also detected in normal cells. Targeting these neoantigens would also influence normal cells, which may lead to serious side effects. Therefore, it is necessary to control dosing by individualized assessment before treatments.

Instead of targeting AS-derived neoantigens directly, combining immunotherapy with splicing modulations is also a promising treatment solution. Targeting the splicing regulators can induce global splicing changes to expand peptide diversity, enhance immunogenicity, and increase ICB therapy’s efficacy. However, the increased complexity of tumor transcriptomics may enable the rapid evolution of tumors to develop new carcinogenic characteristics, such as immune evasion and treatment resistance (Jayasinghe et al., 2018; Kahles et al., 2018). Thus, it is crucial to balance the benefits and risks when combining SF modulation with immunotherapy, either by adjusting the dose or the timing of the therapy.

It is difficult to directly target harmful AS derivatives due to the design or delivery of antisense oligonucleotides or small molecule drugs. Only a few compounds that target specific RNA isoforms have shown clinical utility to date (Sheridan, 2021). ASOs are designed to correct splicing errors by binding a reverse complementary sequence in a target pre-mRNA, thereby preventing its interaction with the splicing machinery. However, it is still a challenge to deliver ASOs to tumor lesions. For example, Spinraza, an FDA-approved treatment that corrects the splicing of SMN2 for spinal muscular atrophy, must be administered by direct injection into the spinal column. Besides, there are also small-molecule compounds that can induce targeted RNA degradation to prevent harmful mis-splicings (Umuhire Juru and Hargrove, 2021). The first small-molecule drug is Evrysdi, which also targets and corrects the splicing of SMN2 (Sivaramakrishnan et al., 2017; Sheridan, 2021). It allows oral administration but is relatively more complicated to design than ASOs. Notably, currently these two types of drugs are more commonly used in genetic diseases rather than tumors, which means it still demands extensive efforts to apply these methods in tumor immunotherapy.

In conclusion, we emphasize that alternative splicing presents a promising avenue for immunotherapy. While the current study in this field is still in its early stage, breakthroughs in both bioinformatics algorithms and biological technologies are critical to accelerate the development of AS-based immunotherapies.
